# Progress in experimental models to investigate the in vivo and in vitro antidiabetic activity of drugs

**DOI:** 10.1002/ame2.12442

**Published:** 2024-06-04

**Authors:** Yasodha Krishna Janapati, Sunil Junapudi

**Affiliations:** ^1^ School of Pharmacy & Health Sciences United States International University‐AFRICA (USIU‐A) Nairobi Kenya; ^2^ Department of Pharmaceutical Chemistry Geethanjali College of Pharmacy Keesara India

**Keywords:** animal models, diabetes mellitus type I, diabetes mellitus type II, in vitro and in vivo models

## Abstract

Diabetes mellitus is one of the world's most prevalent and complex metabolic disorders, and it is a rapidly growing global public health issue. It is characterized by hyperglycemia, a condition involving a high blood glucose level brought on by deficiencies in insulin secretion, decreased activity of insulin, or both. Prolonged effects of diabetes include cardiovascular problems, retinopathy, neuropathy, nephropathy, and vascular alterations in both macro‐ and micro‐blood vessels. In vivo and in vitro models have always been important for investigating and characterizing disease pathogenesis, identifying targets, and reviewing novel treatment options and medications. Fully understanding these models is crucial for the researchers so this review summarizes the different experimental in vivo and in vitro model options used to study diabetes and its consequences. The most popular in vivo studies involves the small animal models, such as rodent models, chemically induced diabetogens like streptozotocin and alloxan, and the possibility of deleting or overexpressing a specific gene by knockout and transgenic technologies on these animals. Other models include virally induced models, diet/nutrition induced diabetic animals, surgically induced models or pancreatectomy models, and non‐obese models. Large animals or non‐rodent models like porcine (pig), canine (dog), nonhuman primate, and Zebrafish models are also outlined. The in vitro models discussed are murine and human beta‐cell lines and pancreatic islets, human stem cells, and organoid cultures. The other enzymatic in vitro tests to assess diabetes include assay of amylase inhibition and inhibition of α‐glucosidase activity.

## Graphical abstract

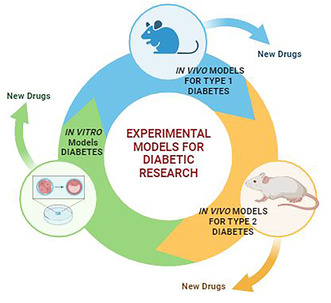

## 1 INTRODUCTION

Diabetes mellitus (DM), a noncommunicable, long‐term, degenerative metabolic disease, has become a serious health issue for the global population. Chronic hyperglycemia is a feature of DM. The primary cause of type 1 diabetes, observed mainly in children, is the loss of pancreatic beta cells.^1^ Insulin resistance and a failure of the beta‐cells to compensate are the two main contributing factors to type 2 diabetes, found mainly in obese people.^2^ Diabetes is also seen in lean people, where it is known as fibrocalculous pancreatic diabetes and classified as type 3 diabetes.^3^ The long‐term effects of DM include cardiovascular problems, retinopathy, neuropathy, nephropathy, and vascular alterations in both macro and microblood vessels.^4^ The International Diabetic Federation has estimated that 1 in 10 adults, or 537 million people globally, have diabetes. According to their projections, there will be 643 million adults worldwide who have diabetes by 2030, and 784 million, that is, 1 in 8 individuals, by 2045. In 2021, diabetes contributed to 6.7 million fatalities, or 1 every five seconds. Over 240 million patients with diabetes are believed to go undiagnosed. Global health spending on diabetes was predicted to be USD 966 billion in 2021 (a 316% increase over the previous 15 years).^5^, ^6^, ^7^ The two countries with the greatest prevalence of the disease are China with 141 million people^8^ and India with 77 million people.^9^ Diabetic people are more likely to contract the virus COVID‐19 and are likely to experience more significant complications. Patients with comorbid conditions like diabetes and heart disease are more likely to experience problems arising from the recent world‐wide COVID‐19 epidemic.^10^

Diabetes poses an important threat to people's health and burdens society financially,^6^ and one of the most popular areas of research currently is the management and treatment of DM. In particular, appropriate animal and advanced in vitro research is crucial for the establishment of innovative, efficient methods of treating conditions like diabetes.^11^ More generally, use of animal models helps researchers create more effective treatments for many disorders and diseases. Humans and other mammals share many biologically related organs, including the heart, lungs, kidneys, liver, and other organs. They are genetically quite similar as well. For instance, the genes of mice and humans are almost identical.^12^ All new medications must first pass legal testing on rodents (often mice or rats) and a bigger nonrodent mammal (typically a dog, pig, or monkey) before being administered to humans. This is done because unfavorable effects in either species frequently point to comparable reactions in people, and if a dose is toxic in both rodent and nonrodent species, it is probably also going to be toxic in people.^13^ However, recently the FDA changed the legislation originally passed in 1938 on animal studies to state that they “no longer require drugs to be tested on animals”.^14^

## 2 METHODOLOGY

This review article is based on the databases PubMed, Cochrane, Virtual Health Library, High Wire, Science Direct, Web of Science, Elsevier, Wiley, and academic Google, etc. The databases were systematically searched for articles published in English from 1922 to 2023 with keywords like diabetes animal models, genetically modified rodent models, chemically induced models, surgical induced models, nonrodent models for type II diabetes, diabetic animal models like canine (dog), porcine (pig) models, feline (cat), obese rhesus monkey, virally induced diabetic type I animal models, transgenic/knock‐out diabetic type I animals, and the cell line models.

## 3 OBJECTIVE

In this this review article, we discuss diabetes complications, diabetes around the world, and diabetes models, including in vivo models and in vitro models for DM.

## 4 ANIMAL MODELS FOR DIABETIC RESEARCH

To accomplish diabetic research, scientists have relied on animal models. Pioneering animal studies on DM in dogs were conducted by Nobel laureates Ivan Pavlov, Fedrick Banting, and Charles Best early last century.^15^ Recently small animals like rodents (mice and rats) are more often exploited for diabetic research,^16^, ^17^ with the ability to delete or overexpress a specific gene by knockout and transgenic technologies making them popular models.^18^ Large animals like porcine (pig) models,^19^ canine (dog) models,^20^ and nonhuman primate models,^21^ as well as Zebrafish models^22^ are outlined in Table 1.

**TABLE 1:** Animal models of type 1 and type 2 DM.

Animal models	Type 1 DM (non‐obese models)	Type 2 DM (obese models)
Chemically induced	Streptozotocin (STZ) Alloxan (ALX) Ferric nitrilotriacetate Dithizone	Gold thioglucose (GTG) treated obese mice
Genetically derived or spontaneous diabetic animals	Rodent models NOD (non‐obese diabetic) mouse BB (Bio Breading) rat LETL (Long‐Evans Tokushima Lean) rat KDP (Komeda diabetes‐prone) rat Lewis‐IDDM (Lewis‐insulin dependent diabetes mellitus) rat Non‐rodent models New Zealand rabbit Keeshond dog Chinese hamster Macaca nemestrina Fascilularis Nigra papio hamadryas	Rodent models ob/ob (obese) mouse db/db mouse KK (Kuo Kondo) mouse KK/Ay (Kuo Kondo/Ay) mouse NZO (New Zealand Obese) mouse NONc/New Zealand obese 10 mouse TSOD (Tsumara Suzuki Obese diabetes) mouse M16 mouse Zucker fatty rat ZDF (Zucker diabetic fatty) rat WDF (winter fatty) rat Non‐rodent models Obese rhesus monkey Feline (cat) Non‐obese models Goto kakizaki (GK) rats Cohen diabetic rat (CDR) Spontaneously Diabetic Torii (SDT) rat Alloxan susceptible Leiter mouse (ALS/Lt) Alloxan‐resistant Leiter mouse (ALR/Lt) Human Islet Amyloid Polypeptide (hIAPP) mice
Transgenic/knock‐out diabetic animals	Insulin receptor substrate‐1,2, glucose transporter‐4, peroxisome proliferator activated receptor knockout mouse Glucokinase knockout mouse	Beta‐3 receptor knockout mouse Uncoupling protein (UCP1) knockout mouse
Other models	Virally induced Coxsackie B virus Encephalomyocarditis virus Kilham rat virus Lymphocytic choriomeningitis virus (LCMV) under insulin promoter Rubella Mumps virus Surgical induced or pancreatectomy Non‐rodent animals like pigs dogs^23^, ^24^ and primates^24^, ^25^ had hyperglycemia after having a pancreatectomy	Diet or nutrition induced diabetic animals C57/BL 6J mouse Desert gerbil Sand rat Spiny mouse Nile grass rat

### 4.1 In vivo models for type 1 diabetes

Type 1 diabetes is a condition involving beta cells in the pancreas,^1^ and therefore diabetic models are created using chemical induction,^26^, ^27^ genetically derived or spontaneously diabetic animals,^28^ or genetically or virally induced animals,^29^, ^30^ in which the functions of pancreatic beta cells in the experimental animals are ultimately destroyed or modified, eventually leading to hyperglycemia, weight loss, hyperphagia etc.^31^, ^32^

#### 4.1.1 Chemically induced diabetes type 1 model

Chemical agent‐induced diabetes in lab animals is the most prevalent option. Among the agents used are streptozotocin (STZ) and alloxan (ALX), both of which achieve a rapid outcome, resulting in an experimental model useful for elucidating the causes of human DM.^17^, ^33^, ^34^ The toxic effects are only specific to pancreatic beta cells, other organs are spared, mortality is low and doses of these diabetogens are specified and have been optimized by many researchers.^26^, ^35^ Due to the rapid rate of beta cell regeneration, therapy is less durable and reversible.^36^ The details of these chemicals such as chemical structure, IUPAC naming, chemical properties, mechanism of action, etc. are given in Table 2. Chemically induced experimental models are frequently chosen to test new diabetes medications and insulin formulations.^37^, ^38^, ^39^ Other diabetogens used in experimental models are dithizone,^40^ cyclosporine, tacrolimus,^41^ dehydroascorbic acid, dehydroisoascorbic acid,^42^ sodium diethyl dithiocarbonate,^43^ potassium xanthate, uric acid, and lithium.^44^

**TABLE 2:** Correlation between alloxan and streptozotocin.^34^, ^45^, ^46^, ^47^, ^48^, ^49^, ^50^, ^51^

	Alloxan (ALX)	Streptozotocin (STZ)
Basic structure	Pyrimidinetrione	d‐Glucopyranose
Chemical structures	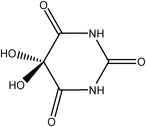	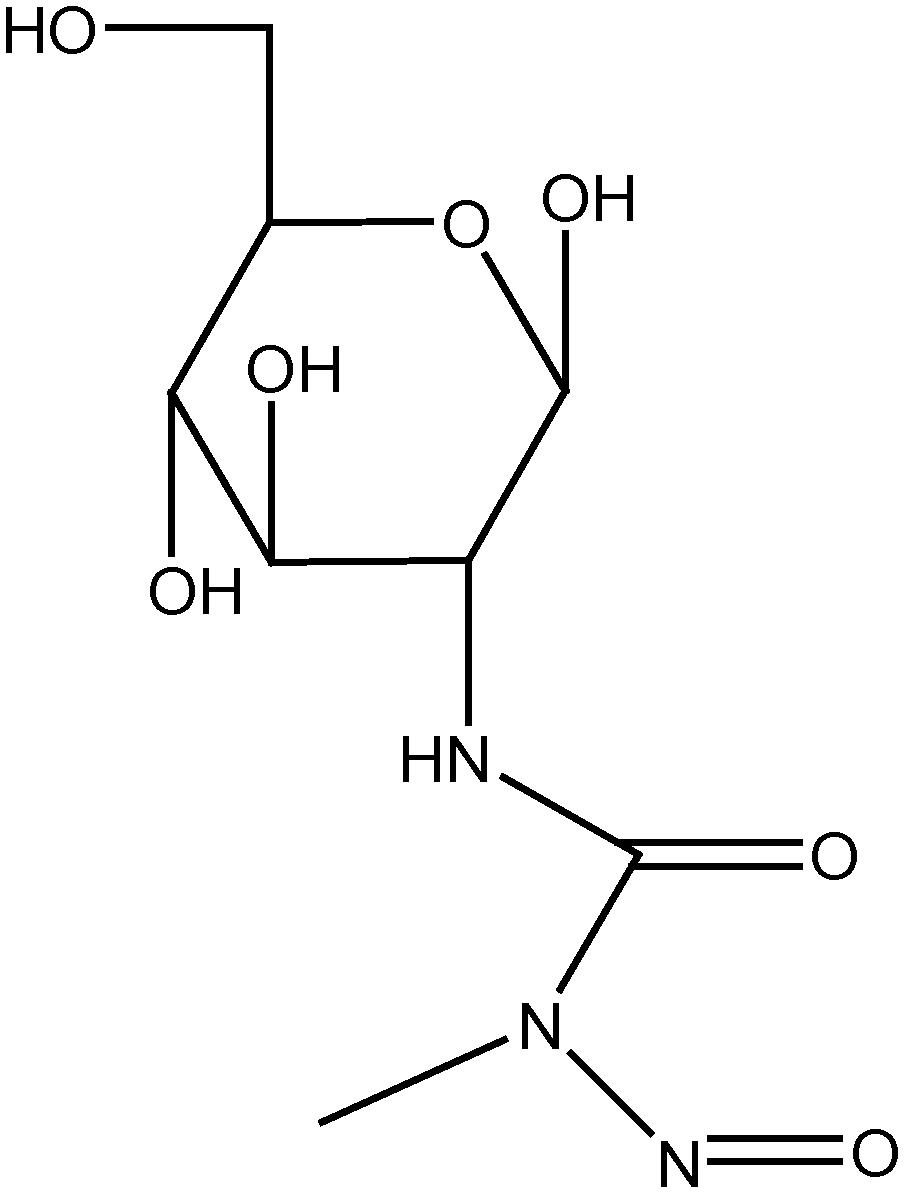
IUPAC name	5,5‐Dihydroxypyrimidine‐2,4,6(1H,3H,5H)‐trione	3‐(Tetrahydro‐2,4,5‐trihydroxy‐6‐(hydroxymethyl)‐2H‐pyran‐3‐yl)‐1‐methyl‐1‐nitrosourea
Chemical properties	Very hydrophilic, beta cell‐toxic glucose analogue (partition coefficient −1.8); weak acid. Chemically unstable (*t* ^1/2^ of 1.5 min at pH 7.4 and 37°C, decomposing to alloxanic acid); stable at acid pH	Hydrophilic, beta cell‐toxic glucose analogue. Relatively stable at pH 7.4 and 37°C (at least for up to 1 h). Stable for 3 years if stored in refrigerator
Route of administration	Subcutaneous (SC)/intravenous (IV)/intraperitoneal (IP)	Subcutaneous (SC)/intravenous (IV)/intraperitoneal (IP)
Dose (mg/kg)	Rat 40–200 Mice 50–200 Rabbit 100–150 Dog 50–75	Rat 35–65 Mice 100–200 Rabbit 65 mg Dog 20–30
Mechanism of action	β Cell toxicity by free radical generation	β Cell toxicity by alkylation process
Acute hyperglycemia	45 min	120 min
Depletion of glycogen	Faster	Slow
Hypoglycemia	Less severe	More severe
Sensitivity to insulin	Yes	Yes
Reversibility	After 3 months	Irreversible
Mortality rate	37%	8%

#### 4.1.2 Genetically derived or spontaneous diabetic type 1 animals

The most commonly used animals for genetically derived type 1 DM are NOD mouse,^52^ BB rat,^53^ LETL rat,^54^ KDP rat,^54^ and LEW‐IDDM rat.^55^ Other animal models less frequently used are New Zealand rabbit,^56^ Keeshond dog,^57^ Chinese hamster,^58^ and different monkeys such as *Macaca nemestrina*, *Fascicularis*, and *Nigra papio hamadryas*.^59^ Genetic mutations that are naturally occurring frequently exhibit an isomorphic phenotypic resemblance between the diabetic animal and the diabetic person, and animals with these mutations are utilized in DM research.^60^ The contrast between the more frequently used animals and humans are detailed in Table 3.^29^, ^44^, ^54^ These animal models are generally monogenic and demonstrate distinct mechanisms of action, whereas the human ADME system is much more complicated.^61^, ^62^ In addition, these animal models are naturally rare and post‐diabetes care aimed at maintaining the animals' health is difficult.^63^, ^64^

**TABLE 3:** Characteristics of difference between humans and genetically derived or spontaneous diabetic animals.

Characteristics	Humans	NOD mouse	BB rat	KDP rat	LEW‐IDDM rat
Diabetes development	Adolescence	24–30 weeks	8–16 weeks	12–16 weeks	8–12 weeks
MHC associated gene	Human leukocyte antigen—DR, DQ isotype	Unique I‐Ag7	At least RT1 B/Du haplotype	At least RT1 B/Du haplotype	At least RT1 B/Du haplotype
Changes in the animal	Hyperglycemia, ketoacidosis	Hyperglycemia and leukocytic invasion of the pancreatic islet of Langerhans are characteristics of the polygenic type 1 diabetes model	Promptly undergo hyperglycemia and ketoacidosis	Spontaneous animal model with nonsense mutation in the Cblb and is a model of autoimmune type 1 diabetes	Develops insulin‐dependent autoimmune diabetes on its own because of pancreatic cell death

#### 4.1.3 Transgenic/knock‐out diabetic type 1 animals

Powerful techniques for determining the role of particular genes in glucose metabolism and the etiology of diabetes include knock‐out and transgenic mice.^29^ Pronuclear microinjection produces transgenic animals that often overexpress the transgene, while gene targeting produces animals with an endogenous target gene deleted or altered (knockout/knockin).^65^ This method can elucidate which transcription factor is responsible for pancreatic development and the signaling pathways of insulin.^66^, ^67^, ^68^, ^69^, ^70^ The various animals in this category include insulin receptor substrate‐1,2 (IRS‐1,2) knockout mouse,^71^ glucose transporter‐4(GLUT4) knockout mouse,^25^ peroxisome proliferator activated receptor (PPAR) knockout mouse,^72^ and glucokinase knockout mouse.^73^

#### 4.1.4 Virally induced diabetic type 1 animal models

Type 1 diabetes development has been attributed to viral infections.^30^ Consequently, beta‐cell destruction has been initiated using viruses in several animal models. Direct infection of the beta cell or the start of an autoimmune reaction against the beta cell can both result in destruction.^74^ The various viruses used to induce DM are Coxsackie B virus,^75^ encephalomyocarditis virus,^76^ Kilham rat virus,^77^ lymphocytic choriomeningitis virus (LCMV) under insulin promoter,^78^ rubella,^79^ and the mumps virus.^80^

The virus‐induced approach can be challenging because the result depends on the virus replicability as well as the time of the infection.^29^ Indeed, research has revealed that, depending on the circumstances, viruses can both cause and prevent autoimmunity.^81^ While viruses have been connected to some type 1 diabetes cases in humans, the extent of the role they play in the disease's development is unknown.^30^, ^82^

#### 4.1.5 Surgically induced models (SIM) or pancreatectomy type 1 diabetic animal models

Non‐rodent animals like pigs,^83^, ^84^ dogs,^23^, ^24^ and primates^24^, ^25^ have hyperglycemia after having a pancreatectomy. This model is a trusted way to cause hyperglycemia when a highly skilled and qualified surgeon is involved. However, the animal undergoes a fairly invasive procedure that raises the risk of hypoglycemia and causes pancreatic exocrine insufficiency.

### 4.2 In vivo models for type 2 diabetes

Insulin resistance and the beta cell's failure to produce insulin to compensate are hallmarks of type 2 diabetes.^2^ Consequently, types of animal models for type 2 diabetes include models of beta cell loss and/or insulin resistance.^44^, ^50^, ^51^ Obesity is prevalent in animal models of type 2 diabetes, mimicking the human scenario where obesity is directly associated with the development of type 2 diabetes.^29^

#### 4.2.1 Genetically derived or spontaneous diabetic type 2 animals (obese model)

The most commonly used animals for type 2 DM are ob/ob (obese) mouse,^85^ db/db mouse,^86^ KK (Kuo Kondo) mouse,^87^ KK/Ay (Kuo Kondo/Ay) mouse,^88^ NZO (New Zealand Obese) mouse,^89^ NONc/New Zealand obese 10 mouse,^90^ TSOD (Tsumara Suzuki Obese diabetes) mouse,^91^ M16 mouse,^92^ Zucker fatty rat,^93^, ^94^ ZDF (Zucker diabetic fatty) rat,^95^ and WDF (Wistar diabetic fatty) rat.^96^ In the above models, development of diabetes is spontaneous and shares many characteristics with typical human type 2 DM. The majority of inbred animal models are homogeneous and under environmental control, which makes genetic analysis simple. Minimum outcome variability necessitates a small sample size.^18^ The characteristics of some of these animals are described in Table 4.

**TABLE 4:** Characteristics of genetically derived or spontaneous diabetic animals (obese model).

Animal	Diabetes development	Cause of DM	Physiological changes
ob/ob (obese) mouse^85^	3–4 weeks	Leptin deficiency	Hyperinsulinemia or insulin resistance, hyperglycemia, hyperlipidemia, obesity
db/db mouse^86^	4–8 weeks	Leptin deficiency	Hyperinsulinemia or insulin resistance, hyperphagic, obesity
KK (Kuo Kondo) mouse^87^	4–5 months	Antagonizing the melanocortin receptor 4 (MCR4) mouse	Hyperinsulinemia or insulin resistance, obesity
NZO (New Zealand obese) mouse^89^	9–12 weeks	Leptin resistance	Hyperinsulinemia or glucose tolerance, insulin resistance, hyperphagic, obesity
TSOD (Tsumara Suzuki obese diabetes) mouse^91^	2 months	Impaired GLUT4 translocation	Polydipsia, polyuria, hyperinsulinemia or insulin resistance, hypertrophy of pancreatic cells, obesity
M16 mouse^92^	3–6 weeks	Heper leptin	Hyperinsulinemia, weight gain, hyperleptinemia and hypercholesterolemia
Zucker fatty rat^93^, ^94^ and ZDF (Zucker diabetic fatty) rat^95^, ^96^, ^97^, ^98^	4 weeks	Defect in leptin receptor signaling	Hyperinsulinemia, hyperlipidemia, glucose tolerance, hypertension, proteinuria, and renal failure

#### 4.2.2 Genetically derived or spontaneous diabetic type 2 animals (non‐obese model)

Lean animal models of type 2 diabetes must also be explored because not all people with DM type 2 are obese. These include models with inadequate beta cells, which eventually results in overt type 2 diabetes in humans (Table 5).^99^, ^100^ These models, which include Goto Kakizaki (GK) rats,^101^ Cohen diabetic rat (CDR),^102^ spontaneously diabetic torii (SDT) rat,^102^ Alloxan susceptible/Lt mouse,^103^ human islet amyloid polypeptide (hIAPP) mice are rare.^104^

**TABLE 5:** Characteristics of genetically derived or spontaneous diabetic animals (non‐obese model).

Animal	Diabetes development	Cause of DM	Physiological changes
Goto Kakizaki (GK)^101^ rats	2–8 weeks	Inadequate pancreatic growth factors and compromised insulin sensitivity in the liver, skeletal muscle and adipose tissues	Hyperglycemia, retinopathy, nephropathy, decreased immune markers^105^
Cohen diabetic rat (CDR)^102^	2 months	Diet changes, reduced insulin secretion	Retinopathy, nephropathy, reduced fertility, testicular degeneration,^106^ hyperglycemia can be retrieved by adjusting diet^39^
Spontaneously Diabetic Torii (SDT) rat^107^	20 weeks	Insulin resistance	Hyperinsulinemia or insulin resistance, ocular issues such as cataract, retinopathy,^108^ gastropathy^109^
Alloxan susceptible/Lt mouse it is used to study both DM I & II^103^	6–8 weeks	Free radical stress	Hyperinsulinemia, impaired glucose tolerance^103^

#### 4.2.3 Diet/nutrition induced diabetic type 2 animals

In these animal models, diabetes is not induced by chemicals or by genetic changes.^12^ Due to insufficient islet compensation, high fat intake can result in obesity, insulin resistance, and impaired glucose homeostasis.^110^, ^111^ Examples of animals in this category are C57/BL 6J mouse, desert gerbil or sand rat,^12^, ^112^ spiny mouse, and Nile grass rat. The characteristics of these animal models are given in Table 6.

**TABLE 6:** Characteristics of diet/nutrition induced diabetic type 2 animals.

Animal	Diabetes development	The diet used to induce DM	Physiological changes
Desert gerbil or sand rat (*Psammomys obesus*).^113^, ^114^	16–24 weeks	High energy nutrition or laboratory chow	Hyperglycemia, ketoacidosis
Spiny mouse (*Acomys cahirinus*).^115^, ^116^	1–2 weeks	High‐energy rodent lab chow	Gain weight and exhibit marked pancreatic beta cell hyperplasia, hypertrophy, increased pancreatic insulin, and ketoacidosis
Nile grass rat^117^, ^118^, ^119^	8–10 weeks	High‐energy rodent lab chow	Obesity, dyslipidemia, hyperglycemia, atherosclerosis, liver stenosis

#### 4.2.4 Non‐rodent models for type 2 diabetic animal models

Non‐rodent animal models includes cats and obese rhesus monkeys. In many ways, feline diabetes mellitus is very similar to human T2DM, including the development of islet amyloid deposits, and complications in a number of organ systems, such as peripheral polyneuropathy and retinopathy.^120^, ^121^, ^122^

The rhesus monkey (*Macaca mulatta*), a non‐rodent model of T2DM, offers the most comparable representation of metabolic problems in diabetes. If kept on an ad libitum laboratory diet, especially fructose, it develops obesity, hyperinsulinemia, and insulin resistance. Over several years, it proceeds to necrosis of beta cells, a sharp drop in insulin levels, and hyperglycemia.^59^, ^123^, ^124^

The Zebrafish model is an attractive model system for the study of metabolic abnormalities. Zebrafish have preserved energy balance and cholesterol metabolism. They are the perfect model for studying lipid metabolism and also, when given an abundance of laboratory nutrients, zebrafish are shown to have hepatic steatosis and higher plasma triglyceride levels. Its fully sequenced genome, ease of genetic manipulation, and greater fertility rates makes it a very versatile model.^125^, ^126^, ^127^, ^128^

## 5 ANIMAL MODELS FOR DIABETIC COMPLICATIONS

Diabetes mellitus is a chronic, sapping metabolic condition that can cause an enormous long‐lasting increase in blood sugar levels. The resulting hyperglycemia plays a key role in the development of diabetic complications, such as damage to organs, both structural and functional, resulting in damage to the kidneys (diabetic nephropathy), eyes (diabetic retinopathy), and nerves (diabetic neuropathy).^129^, ^130^ It is also linked to chronic macrovascular problems such as peripheral vascular disease, coronary heart disease, and stroke (diabetic cardiomyopathy). It has also been discovered that the primary mechanism behind the pathogenesis of such diabetic complications is the generation of oxygen free radical species (ROS).^11^, ^131^ The animal models that are used to analyze these complications are listed in Table 7, along with their characteristics.

**TABLE 7:** Experimental models for diabetic complications.^11^, ^129^, ^130^, ^131^

Diabetic complications	Animal model	Characterization
Diabetic nephropathy^129^, ^130^, ^131^, ^132^	Aldose reductase (ALR2) knockout mice (*Aldor1*−/−)	Development of polyuria, polydipsia and diabetes insipidus
	BB rat	Enhanced GFR, thickening of glomerular basement membrane (GBM)
	C57BL/6	Albuminuria and reduced renal function
	Fat‐fed STZ rat	Albuminuria and pathological changes
	Fructose‐fed rats	Arteriolopathy, renal hypertrophy and glomerular hypertension
	GK rat	Thickening of glomeruli leading to glomerular hypertrophy
	Goto‐Kakizaki (GK)	Glomerular hypertrophy, GBM thickening. Segmental glomerulosclerosis, tubulointerstitial fibrosis
	NOD mice	Enlarged glomeruli and mesangial sclerosis
	MKR mice	Increased GFR, exhibit significant albuminuria
	Zebrafish	Overexpression of CIN85/RukL causing edema
	Zucker diabetic fatty rat	Glomerulosclerosis, tubulointerstitial fibrosis and renal hypertrophy
Diabetic retinopathy^130^, ^133^, ^134^, ^135^, ^136^, ^137^	Alloxan induced model	Microaneurysms with increased acellular capillaries
	Akita mice	Decreased number of amacrine and ganglion cells
	AR deficient (AR−/−) mice	Retinal cell necrosis by leukocytosis.
	Diabetic Torii rats	Retinal thickening, Increased retinal leukostasis, massive hemorrhage.
	db/db mouse	Reduced number of retinal ganglion cells, and thickened retina
	Otsuka Long‐Evans Tokushima fatty rats	Enhanced leukocyte, reduced retinal and retinal nerve fiber layer thickness twisted arteries and veins in the eye
	Wild‐type (WT; C57BL/6J)	Deterioration of retinal capillaries, and elevated generation of superoxide by the retina
	Zucker diabetic fatty rats (ZDF)	BM thickening, loss of endothelial cells (ECs) and pericytes, acellular capillaries, increased capillary hypercellularity
	Zebrafish	Degradation and thinning of the retina
Diabetic neuropathy^130^, ^138^, ^139^, ^140^
	BKS‐db/db Spontaneous	Increased thermal latency, lower tail‐flick response to heat stimulus, decreased sensory nerve fiber velocity, axonal transport, and neurotransmitter levels. Absence of myelinated fiber loss, shrinkage, and breakdown of the myelin sheath
	B6‐ob/ob Spontaneous	Hypoalgesia, tactile allodynia, mechanical response and fiber loss. Increased PARP, immunofluorescence in the sciatic nerve, and spinal cord
	C57BL/6J a diet high in fat	Increased thermal and mechanical latencies, hypoalgesia, hyperplasia allodynia. Peroxynitrite injury in peripheral nerve and dorsal root ganglion neurons
	C57BL/KS (db/db) mice	Decreased sensory nerve conduction velocity and density of intraepidermal nerve fibers (IENF)
	Chinese Hamster	Decreased nerve conduction velocity
	STZ induced rat model	Reduced fiber size of the peroneal nerve and axon than that of the myelin sheath with impaired motor function
Zucker diabetic fatty rats (ZDF)	Reduced motor sensory, and sciatic blood stream. Structural variation in myelinated axons, which causes sensory loss. Elevated nerve sorbitol levels, thermal hyperalgesia
Diabetic cardiomyopathy^141^, ^142^, ^143^, ^144^	Alloxan induced mode	Formation of advanced glycation end products leading to oxidative stress
	BB rats	Reduced calcium—stimulated ATPase activity and cardiac contractility
	GK rats	Hyperglycemia, hyperlipidemia and cardiac cell death
	OLETF rats	Alteration in left ventricular diastolic function
	STZ induced mode	Fibrosis and apoptosis leading to myocardial damage

## 6 IN VITRO TECHNIQUES FOR ASSESSING DIABETES MILLITUS

In vitro (cell or tissue culture)^145^ diabetic models are frequently employed by pharmaceutical companies in the search for new treatments. In vitro models can be employed initially for the screening of test materials or to characterize the cellular or molecular actions of lead chemical substances in advanced phases of development. In vitro models of diabetes are also used in some basic pharmacological research to identify new treatment targets and gain a better understanding of the cellular and molecular mechanisms underlying the illness.^146^ The primary tissues implicated in the pathophysiology of diabetes include the pancreas, liver, muscle, and adipose tissue. These tissues are typically employed to create in vitro models of diabetes used in the drug development process. In vitro models include primary cell cultures generated from normal, diabetic, transgenic animals, or cell lines derived from normal, or transgenic animals.^130^, ^131^, ^147^ The advantages and disadvantages of in vitro models for human diabetes research are specified in Table 8. The other enzymatic in vitro tests to assess diabetes include assay of amylase inhibition and inhibition of α‐glucosidase activity.^148^, ^149^, ^150^ Diabetic complications like diabetic nephropathy, and neuropathy can be assessed by optical fluorescence imaging of the structure of the kidney and nerves. Western blot, ELISA, and PCR can be used to analyze gene expression of inflammatory and oxidative markers. Flow cytometry can be used to investigate the degree of retinal endothelial cell death,^150^ and concurrently it can enumerate overall beta islet cell health and beta cell glucose sensitivity.^151^ Insulin secretion can determined by ELISA.^152^ The luciferase base assay^153^, ^154^ and the glucose uptake assay^155^ can be done using radiolabeling methods.^156^, ^157^ Reporter gene assays can identify PPARg and GLUT‐4.^158^

**TABLE 8:** Advantages and disadvantages of in vitro models for human diabetes research.

In vitro model	Advantages	Disadvantages
Murine beta‐cell lines^159^, ^160^	Simple to culture. There are numerous cell types readily available. A good opportunity to research cell physiology and test medications	The mouse cell line can be challenging to select because of differences from humans. Vascularization and cell‐to‐cell contact are absent
Human beta‐cell lines^161^	Simple to culture. Established human beta‐cell lines permit progress in human diabetes research and clinical applicability	Stable human cell lines are hard to make, and there aren't many of them. Genetic flaws are present in the majority of human cell lines. Grow slowly or respond poorly to glucose. Vascularization and cell‐to‐cell contact are absent
Murine pancreatic islets^162^, ^163^	Can be isolated more quickly and inexpensively than human islets. Simple to genetically modify	Human islets have different islet morphology, vascularization, and blood flow to murine pancreatic islets
Human pancreatic islets^164^, ^165^	Maintain the islet structure. Used to study the biology of the human pancreas	Few donors supply. Don't allow long functional studies. Heterogeneity in their characteristics: size, genetics
Human stem cells^166^, ^167^	A renewable source of beta‐cells. Can be genetically modified. Allow longer studies than pancreatic islets	To obtain them, a long and expensive process is needed
Organoid cultures^168^, ^169^	Resemble the diseased organ architecture better than traditional 2D cultures	Don't have vascularization

## 7 CONCLUSIONS

Studying the pathophysiology and clinical aspects of diabetes mellitus in humans is important because 537 million people are suffering from the disease and 240 million people remain undiagnosed. In addition, the health expenditure incurred in 2021 was 966 billion USD and is expected to increase to 1 trillion USD by 2045. To overcome this endemic condition many new drugs have been introduced into the market after testing in both in vivo and in vitro models. These models of diabetes mellitus are very helpful research tools for testing any new synthetic or herbal drug. In this review, we have summarized the development of various models, including those induced by alloxan and streptozotocin, various models using small animal such as rodents, models involving deletion or overexpression of a specific gene using knockout and transgenic animals (immunogenic), and virally induced diabetic models. We also summarize obese and non‐obese models, and diet or nutrition‐induced models, as well as non‐rodent models which are unique for assessing type 2 DM. The Zebrafish model is considered the most appropriate and advanced model for the screening of diabetes and its complications, that is microvascular complications and retinopathy, but it cannot be used for assessing diabetic nephropathy because of primitive renal cells. Diabetes and diabetic neuropathy can be tested best using rodent models due to their similarity structurally, molecularly, and functionally to humans. These models are also the cheapest and mostly easily available, and are easy to handle and maintain compared to other models. The use of large animals like pigs, monkeys, cats, and dogs is considered for pharmacological screening of diabetes induced by chemicals like alloxan, streptozotocin and even by pancreatectomy, but cost, handling and maintenance are some of the issues to be considered. In vitro models like murine and human beta and pancreatic islet cell lines, human stem cells and organoid culture are also discussed, with their advantages and disadvantages. Although no known animal species closely resembles human diabetes, each model serves as a vital tool for research into genetic, endocrine, metabolic, and morphologic changes, and underlying etiopathogenic changes. The study's design will determine the animal model to use. More suitable animal models could be used if the subsets of type 1 and type 2 diabetes are better understood. The wide range of disease manifestations in either type 1 or type 2 diabetes makes it unlikely that one animal model (or one treatment) will fit. Therefore, it is important to be aware of the advantages and shortcomings of current models (in vivo and in vitro) and to conduct studies bearing in mind that no single model captures all the features or symptoms of disease. Selecting the right animal model can yield crucial information about the pathophysiology process underlying the disease, and this review emphasizes the appropriate use of a variety of animal models whenever possible.

## AUTHOR CONTRIBUTIONS

Yasodha Krishna Janapati: Wrote the manuscript, and corresponding author, conceptualization: conceived and designed the experiments, edited the manuscript and analyzed the data. Sunil Junapudi: Conceptualization, conceived and designed the experiments, edited the manuscript, and critically reviewed the article.

## CONFLICT OF INTEREST STATEMENT

The authors declare no conflict of interest.

## ETHICS STATEMENT

Not applicable.
